# Virtual Testing of Seniors: The Feasibility of Research with Online Neuropsychological Tests (FRONT)

**DOI:** 10.1093/geroni/igab046.3446

**Published:** 2021-12-17

**Authors:** Chris Pauley, Linda Yetman, Jody-Lynn Lupo, Penelope Slack, Diane Jacobs, Chris McGibbon, Manuel Montero Odasso, Pamela Jarrett

**Affiliations:** 1 Horizon Health New Brunswick, Saint John, New Brunswick, Canada; 2 Horizon Health Network, Horizon Health Network, New Brunswick, Canada; 3 University of California San Diego, Alzheimer’s Disease Cooperative Study (ADCS), UC San Diego, Alzheimer's Disease Cooperative Study (ADCS), California, United States; 4 University of British Columbia, Vancouver, British Columbia, Canada; 5 University of California San Diego, La Jolla, California, United States; 6 University of New Brunswick, Fredericton, New Brunswick, Canada; 7 Lawson Research Institute, London, Ontario, Canada

## Abstract

Synergic@Home is a feasibility study evaluating the effects of exercise and cognitive interventions for the prevention of dementia in at-risk individuals over age 60. The COVID-19 pandemic changed the study’s methods, with standardized neuropsychological tests needing to be administered virtually. Experience and research into the viability of neuropsychological assessments administered virtually is limited. After receiving permission to adapt the tests for virtual administration, a neuropsychologist, project managers, and research coordinators developed their approach. A PowerPoint presentation using text and visual stimuli from the tests was developed with on-screen instructions for the raters. An iterative development process involved feedback from the team in order to maximize the fidelity of these methods compared to in-person administration. Mock assessments supervised by a neuropsychologist further refined the methods and confirmed rater adherence to standardized procedures. A secure videoconferencing platform meeting privacy requirements was used. Dual monitors for the raters provided instructions on one monitor while stimuli for the participant was on the second monitor. The participant could only see the stimuli. This method of administering neuropsychological assessment, the Feasibility of Research with Online Neuropsychological Testing (FRONT), is being used to evaluate older adult participants in Synergic@Home. Results from this feasibility study may set the stage for new research methodologies and/or clinical evaluations in the future. This project is funded by the New Brunswick Healthy Seniors Pilot Project and the Canadian Consortium on Neurodegeneration in Aging (with grants from Public Health Agency of Canada and the Canadian Institutes of Health Research, with additional funding partners).

